# ABO Blood Group Genotyping by Real-time PCR in Kazakh Population

**DOI:** 10.5195/cajgh.2014.177

**Published:** 2014-12-12

**Authors:** Pavel Tarlykov, Daniyar Raiymbek, Elena Zholdybayeva, Erlan Ramanculov

**Affiliations:** National Center for Biotechnology, Astana, Kazakhstan

**Keywords:** genotyping, real-time PCR, blood groups, Kazakh population

## Abstract

**Introduction:**

ABO blood group genotyping is a new technology in hematology that helps prevent adverse transfusion reactions in patients. Identification of antigens on the surface of red blood cells is based on serology; however, genotyping employs a different strategy and is aimed directly at genes that determine the surface proteins. ABO blood group genotyping by real-time PCR has several crucial advantages over other PCR-based techniques, such as high rapidity and reliability of analysis. The purpose of this study was to examine nucleotide substitutions differences by blood types using a PCR-based method on Kazakh blood donors.

**Methods:**

The study was approved by the Ethics Committee of the National Center for Biotechnology. Venous blood samples from 369 healthy Kazakh blood donors, whose blood types had been determined by serological methods, were collected after obtaining informed consent. The phenotypes of the samples included blood group A (n = 99), B (n = 93), O (n = 132), and AB (n = 45). Genomic DNA was extracted using a salting-out method. PCR products of ABO gene were sequenced on an ABI 3730xl DNA analyzer (Applied Biosystems). The resulting nucleotide sequences were compared and aligned against reference sequence NM_020469.2. Real-time PCR analysis was performed on CFX96 Touch™ Real-Time PCR Detection System (BioRad).

**Results:**

Direct sequencing of ABO gene in 369 samples revealed that the vast majority of nucleotide substitutions that change the ABO phenotype were limited to exons 6 and 7 of the ABO gene at positions 261, 467, 657, 796, 803, 930 and 1,060. However, genotyping of only three of them (261, 796 and 803) resulted in identification of major ABO genotypes in the Kazakh population. As a result, TaqMan probe based real-time PCR assay for the specific detection of genotypes 261, 796 and 803 was developed. The assay did not take into account several other mutations that may affect the determination of blood group, because they have a low occurrence rate and therefore have not been found in the population sample.

**Conclusion:**

Real-time PCR based method for fast and reliable ABO genotyping was developed. This assay may be used as a complement to classic serological blood typing.

